# Supplementary material for: A Qualitative Study of the Pain Experiences of Children and Their Parents at a Canadian Children’s Hospital
Source: Children (Basel). 2022 Nov 23;9(12):1796. doi: 10.3390/children9121796 (PMC9777277; doi:10.3390/children9121796)
Supplement: Supplementary file 1 [file children-09-01796-s001.zip › children-2002400-supplementary.pdf]

Supplementary Materials
Interview Guide

Note: Follow-up questions as bullets; optional questions in italics

1. Tell me about your/your child's experiences with pain?
Prompt participant that they can share multiple experiences if they choose

- What happened?
    - How long was it happening before you sought help?
    - How long did it last?
- How has it affected you?
    - What comes to mind about your experiences?
- How has the Stollery played a role in this?
    - Does being at the hospital alter your awareness of pain management? If yes, what are some examples of this?
    - Did you need to seek care elsewhere to help or return to the Stollery at a later point?
- *What does pain mean to you?*

2. What did you expect from the Stollery to help manage your/your child's pain?

- For example, with regard to pain medicines, distraction, help from healthcare providers from other disciplines.
- Did you expect that your/your child's pain should have been decreased or eliminated?
- Did you receive support from someone who provided culturally or spiritually relevant supports?
- *What role did being comfortable play (if any) in your/your child's pain management?*

3. What was actually offered to you to help manage your/your child's pain?

- Who offered it, when, and how often?
- Were you/your child given any strategies or resources you could use independently in the hospital or when you returned home?
- Do you feel anything should have been provided by was not?
- *Were you offered it more than once/by a different healthcare provider?*
- *What was your experience regarding each?*
- *Did you/would you feel comfortable asking for it?*
- *Who would you feel most comfortable having provide pain care for you/your child?*

4. How involved did you feel in your/your child's pain management?

- Did you feel comfortable to ask more questions about the pain management plan?
    - Was there something staff did to make you comfortable or uncomfortable asking questions?
    - Did you receive resources or strategies for the future?

- *What did you see as your role in managing your/your child's pain to be?*
- *Did you feel you had a voice in your/your child's pain management plan?*
  - *Was there something staff did/didn't do to make you feel as though you have a voice in your/your child's pain management?*
- *Did you feel pressured to go along with the healthcare provider's suggestions?*

5. Did you receive any resources or materials to explain how to manage your/your child's pain or did you find any through your own research?

- If so:
  - Describe the resource
  - What did you like about it?
  - Who gave it to you?
  - What could be better about it?
- If found through your own research:
  - Describe the resource
  - How did you find it?
  - Do you think this would be useful to be provided at the Stollery? If so, to whom?
- If not:
  - What would be useful to you?
  - What format would it have? (e.g., pamphlet, video, link to a webpage)

6. If you could describe your experience with your/your child's pain management at the Stollery in 3 words, what would those words be?

7. Do you think managing pain is important at the Stollery?

- What would be your expectation of pain management if you had to go to the Stollery again at a later time?
- What could we do better?
  - Create a list of 1-3 things with a ranking, if participant is unsure how to answer
- What did we do best?
  - Create a list of 1-3 things with a ranking, if participant is unsure how to answer
